# Impacts of florfenicol on the microbiota landscape and resistome as revealed by metagenomic analysis

**DOI:** 10.1186/s40168-019-0773-8

**Published:** 2019-12-09

**Authors:** Qifan Zeng, Chao Liao, Jeffery Terhune, Luxin Wang

**Affiliations:** 1 Department of Animal Sciences, Auburn University, Auburn, AL 36830 USA; 2 Ministry of Education Key Laboratory of Marine Genetics and Breeding, College of Marine Science, Ocean University of China, Qingdao, 266003 Shandong China; 3 Department of Food Science and Technology, University of California Davis, Davis, CA 95616 USA; 4 Department of Fisheries and Allied Aquacultures, 203 Swingle Hall, Auburn University, Auburn, AL 36849 USA

**Keywords:** Florfenicol, Catfish, Aquaculture, Antimicrobial resistance, Microbiome, Antibiotics, Horizontal gene transfer, Mutation

## Abstract

**Background:**

Drug-resistant fish pathogens can cause significant economic loss to fish farmers. Since 2012, florfenicol has become an approved drug for treating both septicemia and columnaris diseases in freshwater fish. Due to the limited drug options available for aquaculture, the impact of the therapeutical florfenicol treatment on the microbiota landscape as well as the resistome present in the aquaculture farm environment needs to be evaluated.

**Results:**

Time-series metagenomic analyses were conducted to the aquatic microbiota present in the tank-based catfish production systems, in which catfish received standard therapeutic 10-day florfenicol treatment following the federal veterinary regulations. Results showed that the florfenicol treatment shifted the structure of the microbiota and reduced the biodiversity of it by acting as a strong stressor. Planctomycetes, Chloroflexi, and 13 other phyla were susceptible to the florfenicol treatment and their abundance was inhibited by the treatment. In contrast, the abundance of several bacteria belonging to the Proteobacteria, Bacteroidetes, Actinobacteria, and Verrucomicrobia phyla increased. These bacteria with increased abundance either harbor florfenicol-resistant genes (FRGs) or had beneficial mutations. The florfenicol treatment promoted the proliferation of florfenicol-resistant genes. The copy number of phenicol-specific resistance genes as well as multiple classes of antibiotic-resistant genes (ARGs) exhibited strong correlations across different genetic exchange communities (*p* < 0.05), indicating the horizontal transfer of florfenicol-resistant genes among these bacterial species or genera. Florfenicol treatment also induced mutation-driven resistance. Significant changes in single-nucleotide polymorphism (SNP) allele frequencies were observed in membrane transporters, genes involved in recombination, and in genes with primary functions of a resistance phenotype.

**Conclusions:**

The therapeutical level of florfenicol treatment significantly altered the microbiome and resistome present in catfish tanks. Both intra-population and inter-population horizontal ARG transfer was observed, with the intra-population transfer being more common. The oxazolidinone/phenicol-resistant gene *optrA* was the most prevalent transferred ARG. In addition to horizontal gene transfer, bacteria could also acquire florfenicol resistance by regulating the innate efflux systems via mutations. The observations made by this study are of great importance for guiding the strategic use of florfenicol, thus preventing the formation, persistence, and spreading of florfenicol-resistant bacteria and resistance genes in aquaculture.

## Introduction

The global seafood consumption increased from 9.9 kg per capita in the 1960s to 20 kg in 2014, and it is expected to continue increasing in the future [1]. Aquaculture has grown dramatically over recent decades and become an important component of world fish production to satisfy the consumption demands. The production of finfish is now about three quarters of that from wild fisheries and reaches over 73 million tons live weight [1]. Since antibiotics have been widely applied to prevent severe loss due to infectious disease, criticisms and concerns have arisen related to the potential public health risks and environmental interferences caused by aquaculture effluents [2, 3]. As antibiotics cannot be effectively metabolized by aquaculture animals, more than 70% of antimicrobials used in aquaculture enter the environment with intact activity [4, 5]. These antibiotic residues impose selection pressures on aquatic microbes and promote the spread of antibiotic-resistant (AR) bacteria, even at concentrations below the minimum inhibitory concentration (MIC) of susceptible wild-type bacteria [6]. Studies have estimated that 90% of aquatic bacteria are resistant to at least one antibiotic, and approximately 20% are resistant to five or more [7]. In addition, a growing number of microbes are being identified as carrying genes with novel antibiotic-resistant mechanisms [8]. The genetic plasticity of the microbial community enables antibiotic-resistant genes (ARGs) to disseminate throughout aquatic bacterial populations and communities, making aquaculture systems that lack antibiotic restrictions suffer high risks of ARG transmission [2]. Antibiotic residues, along with ARGs and AR bacteria, are disseminated into the environment through aquaculture effluent, which could reduce the therapeutic potential of antibiotics against pathogens and alters the natural bacterial flora [9, 10]. Hence, there is an urgent need to understand how AR pathways spread and evolve in aquaculture systems.

Florfenicol is a fluorinated synthetic analog of chloramphenicol that is exclusively used in veterinary medicine [11]. It has broad-spectrum bacteriostatic activity for a wide range of microorganisms by reversibly binding to the peptidyltransferase center at the 50S ribosomal subunit and thus inhibiting the bacterial protein biosynthesis [12]. In the USA, it was approved by the FDA for treating enteric septicemia of catfish in 2005, coldwater disease in salmonids in 2007, furunculosis in freshwater-reared salmonids in 2007, and the streptococcal septicemia and the columnaris disease in freshwater-reared finfish in 2012 [13]. Unfortunately, the excessive use of florfenicol as an antimicrobial chemotherapeutic agent in animal husbandry potentially promotes the prevalence and abundance of FRGs in surrounding environment [14].

Over the years, studies have revealed a number of novel genes which enable microbials to mitigate the inhibitory effects of florifenicol [15, 16]. The first florfenicol resistant gene (*floR*) was derived from a transferable R-plasmid in *Pasteurella piscicida*, a gram-negative fish pathogen in 1996 [17]. Since then, it has been identified in the chromosome of multi-resistant *Salmonella* Typhimurium DT104, *Vibrio cholerae*, *E. coli*, *Bordetella bronchiseptica*, and *Acinetobacter baumannii*, or on the plasmids of *E. coli*, *Salmonella* Newport, *Klebsiella pneumoniae*, *Pasteurella multocida*, *Bibersteinia trehalosi*, *Actinobacillus pleuropneumoniae*, and *Stenothrophomonas maltophila* [16, 18]. The comparisons of the nucleotide and amino acid sequences of these genes revealed limited homology with the known phenicol-resistant determinants, suggesting that the presence and the diversity of florfenicol-resistant genes (FRGs) in the environment have yet to be adequately evaluated [18]. Other FRGs, such as the exporter genes *fexA* [19], *fexB* [11], and *optrA* [20], as well as the 23S rRNA methyltransferase gene *cfr* [21], have also been identified from different bacterial isolates of various animal origin. Many of the FRGs are located in mobile genetic elements, such as plasmids, transposons, or gene cassettes [11, 20, 22, 23]. Therefore, whether the florfenicol therapy may lead to the emergence of other resistance determinants for multiple drug classes and cause substantial impact on the antibiotic resistome needs to be evaluated as well.

The metagenomic sequencing technology and its associated analysis methods and computational tools have provided new strategy for investigating the resistome of agricultural and environmental microbiota. It has been used for direct detection of ARGs in animal feeding facilities [24], agricultural soils amended with manure [25], and effluent wastewater [26]. Our previous study has revealed that aquaculture waste containing therapeutic antibiotics has a substantial impact on aquatic microbial populations present in the production system and causes selective pressures on AR genes, especially efflux pumps [27]. Thus, to better address the concerns and questions associated with the impact of therapeutic florfenicol treatment on microbiome populations and the resistome present in the aquaculture farming environment, a time-series metagenomic analysis was performed on a model tank-based catfish production system. The aim of this study was to provide an in-depth evaluation of the patterns and dynamics of the microbial resistome in response to florfenicol treatment and illustrate the impact of chemotherapeutic treatment on the aquaculture environment.

## Results

### Microbial community analysis

**Fig. 1:**
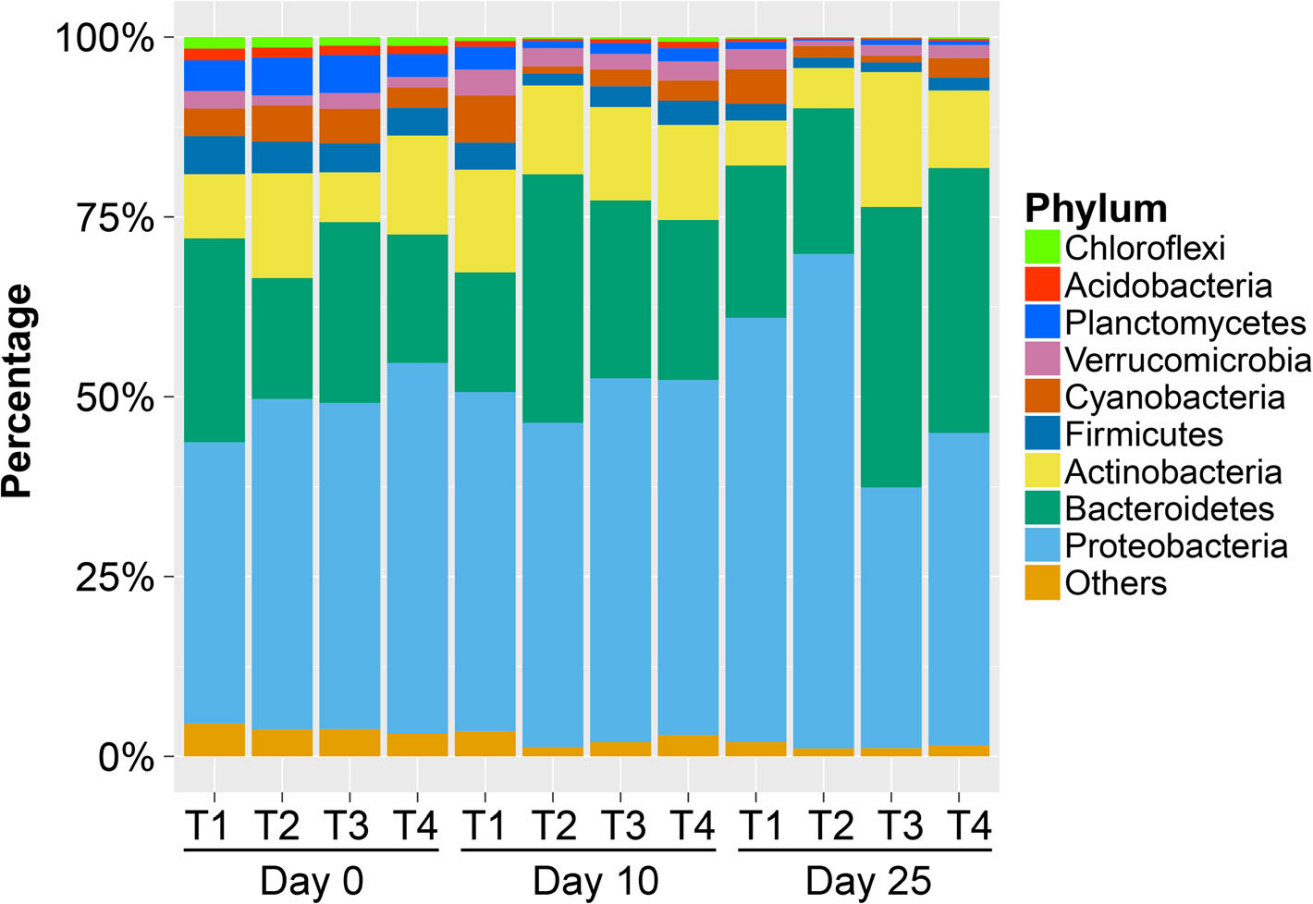
Microbiome composition at the phylum level samples from tank 1 (T1), tank2 (T2), tank 3 (T3), and tank 4 (T4). The top nine phyla were reported for each sample, and all other phyla were grouped into “Other”

Time-series metagenomic analyses were conducted to investigate the changes of aquatic microbiota and resistomes present in the tank-based catfish production system before and after 10 consecutive days of therapeutic florfenicol treatment. Water samples were collected from four replicate tanks on days 0, 10, and 25 (Table 1), and the total DNA was extracted from these water samples for sequencing. A total of 580.04 million reads with an average read length of 100 bp were generated from the 12 samples collected from the treatment tanks. After trimming, a total of 544.51 million filtered reads (93.87%) were retained, resulting in over 7 Gb sequencing data for each sample (Table 1). Taxonomic analysis revealed that 47.83 million filtered reads of the samples from day 0 were assigned to the genus level, accounting for 25.45% of the filtered reads. For the samples from day 10, 50.35 million reads (28.33%) were matched to reference sequences at the genus rank. For samples collected on day 25, 68.98 million reads (38.58%) were annotated at the genus rank. The microbial community compositions in all the samples were similar at the rank of phylum. Over 99% of the total identified species were bacteria, while fewer than 1% were archaea. Proteobacteria was the most abundant bacterial phylum in all the 12 samples, accounting for approximately 50% of the total species. Proteobacteria, Bacteroidetes, Actinobacteria, Cyanobacteria, Firmicutes, Planctomycetes, Verrucomicrobia, Acidobacteria, and Chloroflexi were the nine most abundant phyla. These phyla jointly accounted for over 96% of the total bacteria across all the samples (Fig. 1).

**Table 1:** Summary of Illumina sequencing data and trimming

Sample	Number of raw reads (million)	Read length (bp)	Number of reads after trim (million)	Average length after trim (bp)	Total bases after trim (Gb)
Day 0					
Tank 1	50.67	100	47.8	92.66	8.86
Tank 2	45.03	100	42.41	92.13	7.81
Tank 3	57.55	100	54.17	91.79	9.94
Tank 4	46.44	100	43.58	91.91	8.01
Day 10					
Tank 1	44.15	100	41.29	91.84	7.58
Tank 2	44.73	100	41.87	91.84	7.69
Tank 3	54.67	100	51.25	91.98	9.43
Tank 4	46.16	100	43.34	92.32	8
Day 25					
Tank 1	44.7	100	42.16	92.87	7.83
Tank 2	45.88	100	42.93	92.06	7.9
Tank 3	42.56	100	39.77	91.34	7.27
Tank 4	57.5	100	53.94	92.52	9.98
Total	580.04	–	544.51	–	100.3

**Fig. 2:**
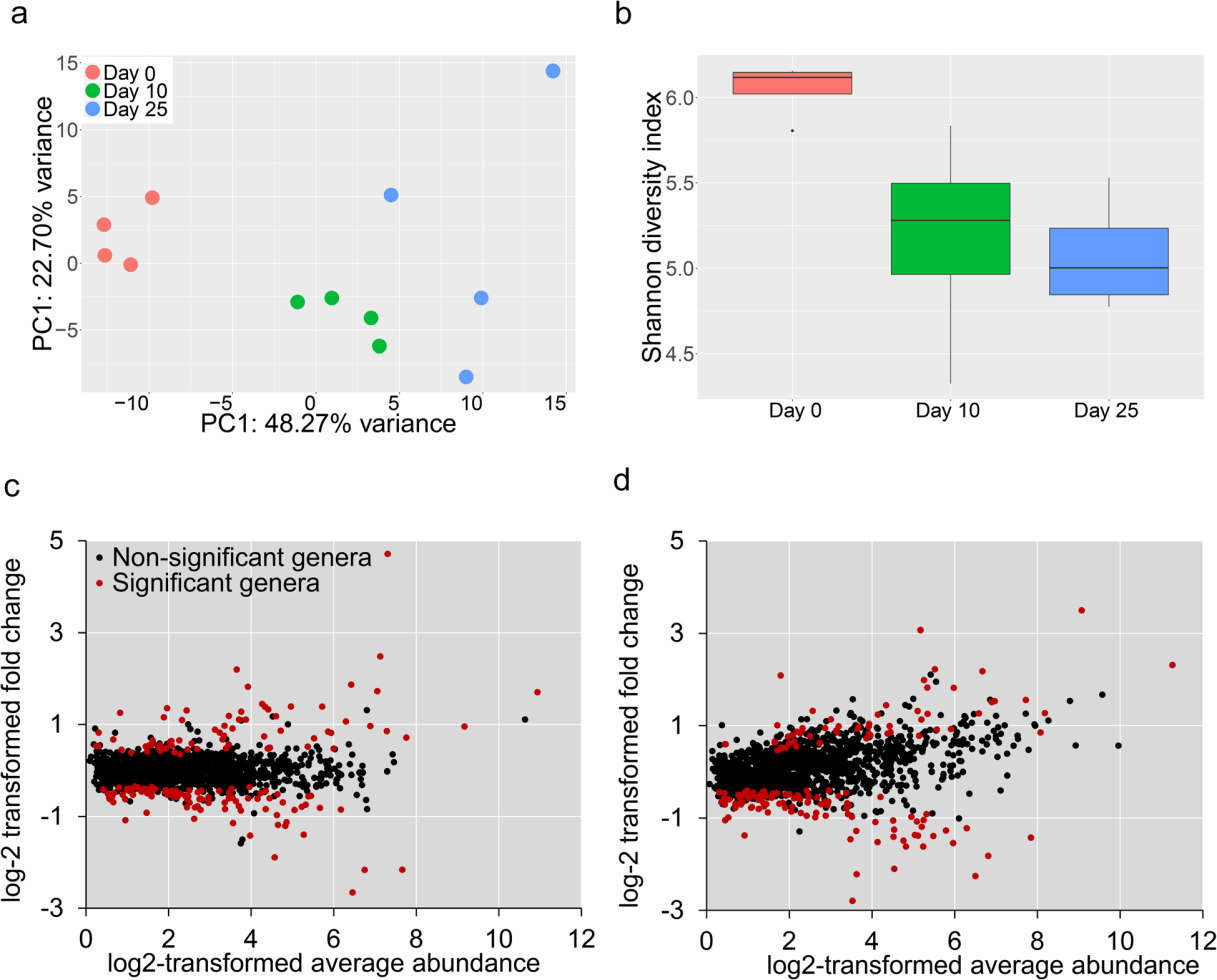
**a** PCA analysis of metagenomic samples. **b** Shannon diversity index of samples from three time points. **c**, **d** Comparisons of bacterial genera abundance (**c** day 10 vs day 0; **d** day 25 vs day 0)

The principal component analysis (PCA) confirmed the relevance of the data: samples from the three groups were separated by the first axis, which explained 48.27% of the species abundance variability (Fig. 2a). Samples from day 0 were separated from the other two groups. The variance within sample groups explained 22.7% of the species abundance variabilities, with the largest within-group variance observed on day 25. The average Shannon diversity index decreased in a time-dependent manner, with the highest value observed on day 0 and the lowest value observed on day 25 (Fig. 2b).

Pairwise comparisons were conducted to check the microbial abundance differences using a zero-inflated log-normal model implemented in the MetagenomeSeq package (FDR-corrected *p* value < 0.05). Phylum Planctomycetes, Acidobacteria, and Chloroflexi were significantly reduced in samples for day 10 and day 25, whereas phylum Proteobacteria, Bacteroidetes, and Verrucomicrobia were significantly increased on day 25 compared to day 0 (Additional file 1: Table S1). No significant difference was observed between samples from day 10 and day 25 at phylum rank. At the rank of genus, a total of 1444 genera were identified across all the samples. Substantial changes were observed on day 10 and day 25 when compared to day 0 (Table 2). When comparing the abundance of genera identified on day 10 with their corresponding abundance on day 0, a total of 170 differentially abundant genera were identified, including 70 genera with increased abundance and 100 genera had decreased abundance (Fig. 2c; Additional file 2: Table S2). When comparing the day 25 with the day 0, the abundance of 165 genera was significantly different from day 0, including 59 genera with increased abundance and 106 genera with decreased abundance (Fig. 2d; Additional file 3: Table S3). Together, a total of 262 differentially abundant microbial genera were identified. Despite that the abundance was similar at the phylum level, 36 genera of Firmicutes, 20 genera of Actinobacteria, six genera of Verrucomicrobia, and four genera of Cyanobacteria exhibited significant changes in abundance. Only 15 differentially abundant archaeal genera were identified, of which 14 Genera were significantly decreased. Notably, microbial compositions of day 10 and day 25 were found to be similar with no significantly altered genus.

**Table 2:** Statistics of differentially abundant genera (FDR *p* values < 0.05) of samples from day 10 and day 25 compared to samples from day 0

Sample	Increased		Decreased		Total
	Bacteria	Archaea	Bacteria	Archaea	
Day 10	69	1	98	2	170
Day 25	59	0	92	14	165

### De novo metagenome assembly and phylogenetic assignment

Bacterial genomes were reconstructed with the combined assembly of filtered reads from all the samples. A total of 2,221,395 contigs with N50 size of 2253 bp were assembled from the pooled sequencing reads. Gene annotation was performed using the JGI pipeline, which identified 763,897 genes from the metagenomics assembly. The genes were functionally categorized using the Pfam, KEGG, and COG databases.

**Fig. 3:**
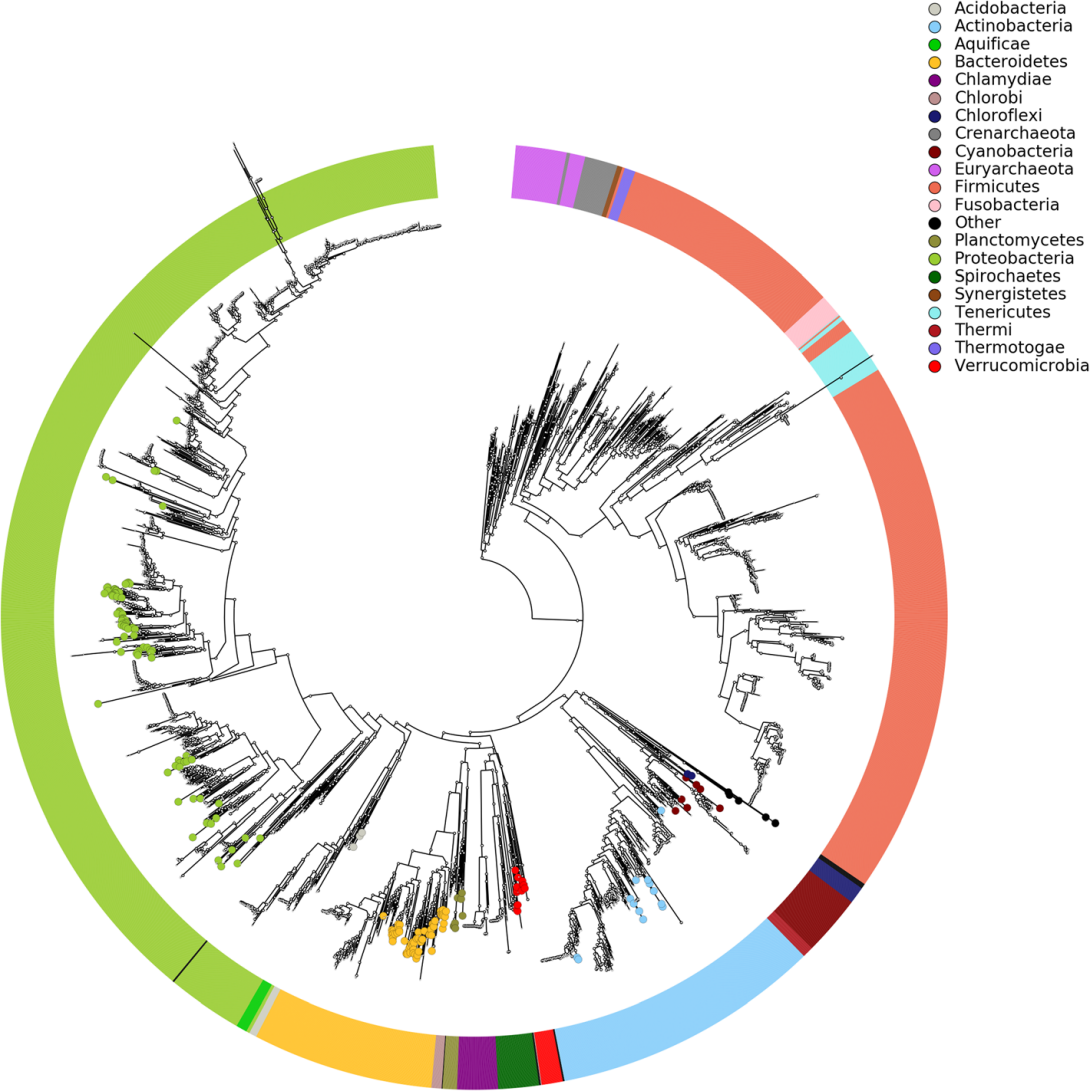
Phylogenetic assignment of assembled genome bins. The phylogenetic tree was obtained with PhyloPhlAn using 400 broadly conserved proteins to extract the phylogenetic signal. Organisms are colored based on phyla

Assembled contigs were organized into 626 genome bins based on tetranucleotide sequence composition and coverage patterns across the samples. After filtering low-quality genome bins, phylogenetic analyses were conducted for the 198 qualified genome bins. The phylogenetic tree revealed that they belonged to nine bacterial phyla, including Candidatus, Saccharibacteria, Chloroflexi, Cyanobacteria, Actinobacteria, Verrucomicrobia, Planctomycetes, Bacteroidetes, Acidobacteria, and Proteobacteria (Fig. 3). As shown in Additional file 4: Table S4, 43 genome bins were classified at the genus and species level, 47 were classified at the family level, 29 were classified at the order level, 15 were classified at the class level, and the remaining 64 were identified at the phylum level because of the limitation of available related reference genomes.

### Analysis of gene frequency changes

**Fig. 4:**
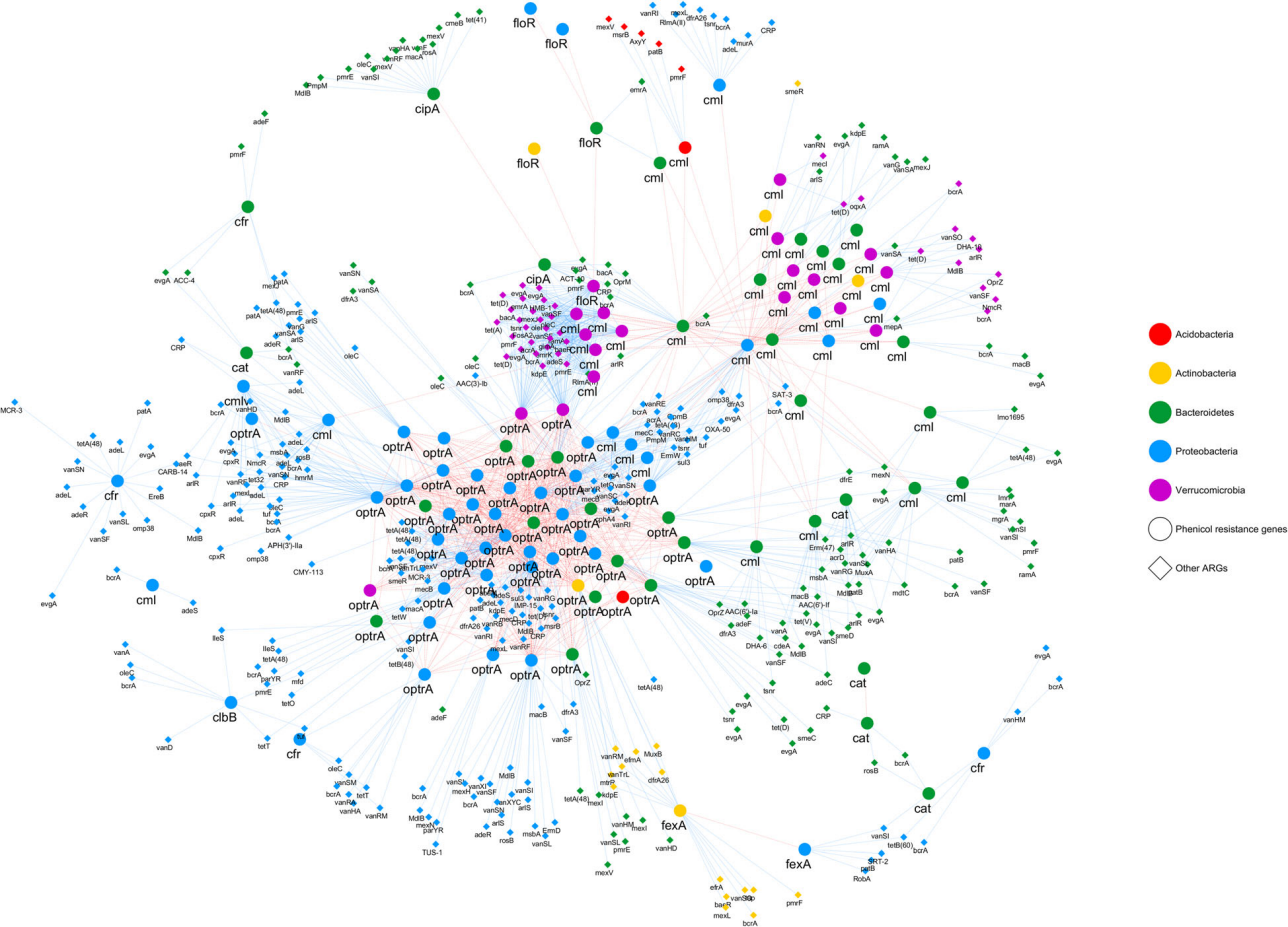
Co-occurrence networks of ARGs. The blue lines represent significant correlations of ARGs within a microbial genome bin, while the red lines represent significant correlations of ARGs between different genome bin populations

To identify potential horizontal gene transfer of ARGs, the patterns of gene copy number variations within metagenomic populations were revealed by the time-series analysis. The relative abundance of phenicol-resistant genes in 59 genome bins was significantly increased on days 10 and 25 compared to day 0 (Additional file 8: Figure S1; Additional file 5: Table S5). Comparison of gene frequency distributions could reveal possible horizontal gene transfer events [28]. Therefore, a co-occurrence network was construct employing the gene frequency data to identify potential ARG transfers involved in florfenicol treatment-stimulated resistome alterations (Fig. 4). As shown in Fig. 4, significant correlations were observed among ARGs that confer resistance to multiple drug classes, including phenicol (*cat*, *cfr*, *cml*, *fexA*, *floR*, *optrA*), aminoglycoside (*AAC (2′)-Ia*, *acrD*, *APH (3′)-IIa*, *smeR*), penam (*CARB-14*, *ACT-10*, *ACT-37*, *mecI*), fluoroquinolone (*abeM*, *emrA*, *emrR*, *evgA*, *mexH*), tetracycline (*tet (41)*, *tet(A)*, *adeF*, *mdfA*), glycylcycline (*vanA*, *vanD*, *vanF*, *vanXM*, *vanXYC*), carbapenem (*BJP-1*, *cphA4*, *mecA*, *adeK,*), diaminopyrimidine (*dfrA26*, *dfrA3*, *dfrD*, *dfrE*), macrolide (*EreB*, *macA*, *AxyY*, *cmeB*, *mtrR*), and rifamycin (*efrA*). Intra-population correlations (indicated by blue edge) were more common than inter-populations correlations which were mainly observed in phenicol resistance genes (indicated by red edge). The oxazolidinone/phenicol-resistant gene *optrA* was identified as the most prevalent transferred ARGs, with the frequency expanded in 50 genome bin populations.

### SNP identification and genetic heterogeneity in sequence-discrete populations

The intra-population genetic diversity of sequence-discrete populations was examined by identifying SNPs within genome bins. By mapping high-quality reads from all time points to the metagenomic assembly, we identified different levels of SNP polymorphism in every genome bin, ranging from 67 SNPs per Mbp in *Bacteroidetes*-262 to 19,090 SNPs per Mbp in *Flavobacterium*-239 (Additional file 6: Table S6). Most genome bins had > 1000 SNPs per Mbp, but seven genome bins had < 100 SNPs per Mbp, including *Alcaligenaceae*-300, *Cyanobacteria*-321, *Methylophilaceae*-222, and four genome bins from phylum Bacteroidetes (*Bacteroidetes*-185, *Bacteroidetes*-203, *Bacteroidetes*-219, *Bacteroidetes*-262). Five of the seven genome bins had relatively lower estimates of genome completeness (> 75%), suggesting that genetic variations within these sequence-discrete populations might be underestimated. However, completeness and coverage depths alone could not account for the large differences in SNP counts among populations. For example, *Flavobacterium*-239 had approximately threefold more SNPs than its closely related phylogenetic group *Flavobacterium-*99, despite the fact that *Flavobacterium-*99 had a higher level of genome completeness and coverage.

Most of the SNPs (~ 91%) within the genome bin populations were in genic regions, and the remaining ~ 9% were located in intergenic regions. However, over 73% of the SNPs were silent and did not result in amino-acid substitutions, indicating that most of the genetic variation within the microbial populations may be neutral and did not cause competitive exclusion among coexisting genotypes. SNP mutations that generate premature stop codons were observed in 181 of the total 198 genome bins. These mutations result in nonfunctional genes and accounted for only ~ 0.1% of the total SNPs. A small proportion of the identified SNPs (~ 18%) were missense mutations, suggesting that negative selection caused variations to accumulate in most of the microbial populations. By applying the quasibinomial GLMs, SNP allele frequency differences were estimated over time in all populations. The fraction of total SNPs dominated by a single allele was low in most of the populations, suggesting that the overall level of genetic heterogeneity in most populations did not change dramatically. The allele frequency of 4456 SNPs from 15 genome bins shifted consistently when comparing samples from day 10 to samples from day 0. The most dramatic change was observed in the *Microbacteriaceae*-290 population, of which 1434 displayed consistent allele frequency differences, indicating large changes in the relative abundance of different genotypes within these sequence-discrete populations. When comparing the samples from day 25 to samples from day 0, only 645 SNPs exhibited consistent difference across replicates. In spite of the 20 SNPs from three genome bins, the remaining 625 SNPs were from contigs that could not be assigned to genome bins.

**Fig. 5:**
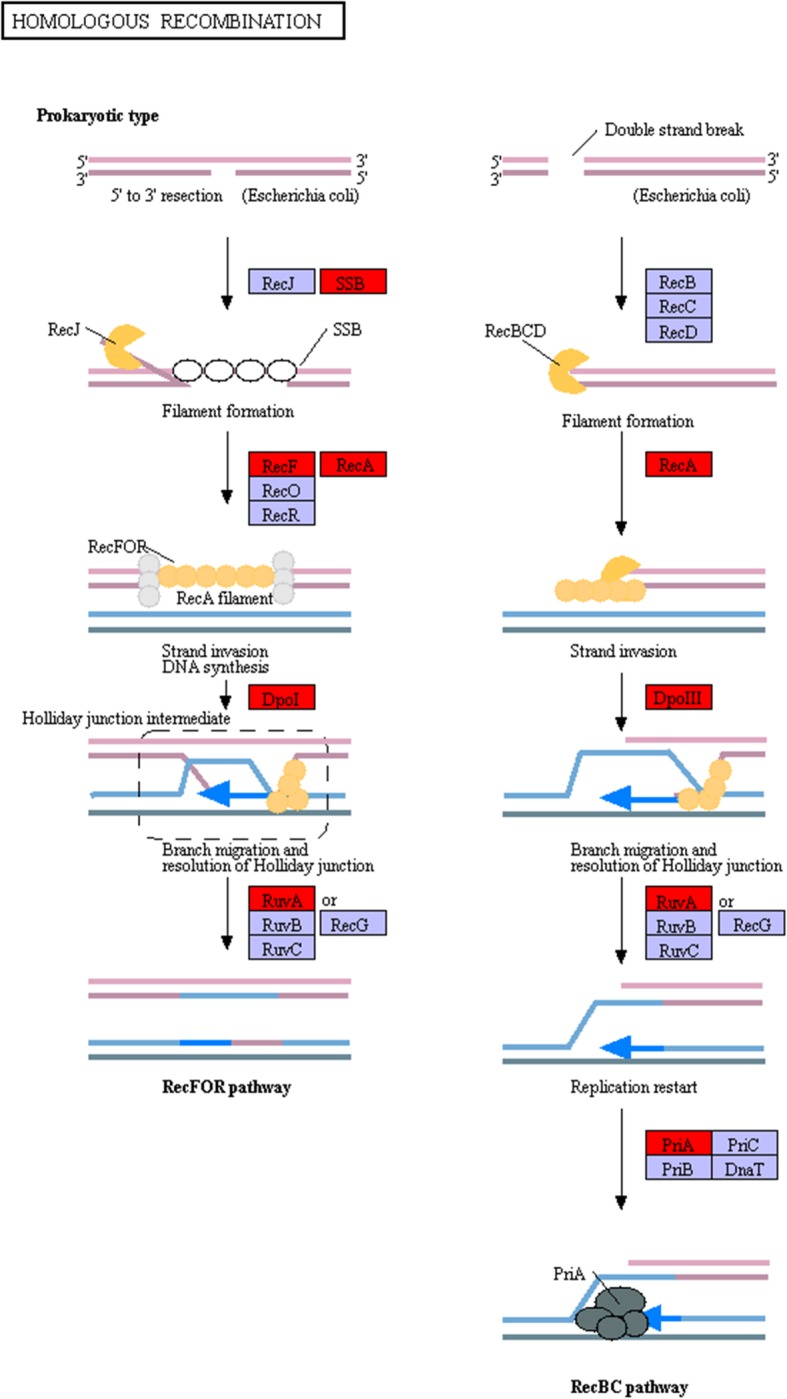
Recombination pathways covered by significant SNPs. Genes covered by SNPs with constant allele frequency changes are denoted by red shading

To understand the effects of these substantial shifts of alleles, genes covered by these SNPs were extracted for functional module annotation and pathway analysis. We found that these mutations were mainly related to transmembrane transport and DNA recombination (Additional file 9: Figure S2). Various transporters, including MFS-family permease, ABC-type transport system permease, drug/metabolite transporter (DMT) superfamily permease, and phosphate-transport-system permease, were identified with significant changes of allele frequencies (Additional file 7: Table S7). Interestingly, multiple key genes participating in homologous recombination (e.g., single-strand DNA-binding proteins, DNA recombinase, DNA polymerase, and ATP-dependent DNA helicase) were covered by the significant SNPs (Fig. 5).

## Discussion

In the USA, only three drugs, including florfenicol, oxytetracycline, and sulfadimethoxine/ormetoprim, have been authorized for bacterial disease treatment in aquaculture [13]. Unfortunately, a few strains of *E. ictaluri* (a fish pathogen that can cause Enteric Septicemia of catfish) had been identified that are resistant to both Romet-30® and Terramycin® 200 [29]. The emergence of these antibiotic-resistant fish pathogens poses critical needs to better understand the formation of resistance, the source of the resistome, as well as the impact and involvement of the aquaculture environment in the formation and transfer of resistome. As an analog of chloramphenicol and thiamphenicol, the C3 position of florfenicol is fluorinated and cannot act as acceptor site for acetyl groups, making florfenicol resistant to inactivation by CAT enzymes [30]. Bacterial resistance to florfenicol is conferred by two main mechanisms, one is through reduced membrane permeability and the other is based on the mutation of the 50S ribosomal subunit. Many of these FRGs and 50S ribosomal mutations are not exclusive for florfenicol resistance; they also confer resistance to phenicol and some structurally unrelated antimicrobial groups, such as lincosamides, oxazolidinones, and pleuromutilins [20, 21]. Therefore, it is of great importance to understand the potential effects of florfenicol on the microbiota landscape and resistome of the farm environment.

In this study, metagenomic analysis revealed that florfenicol administration had a pronounced effect on the composition of the bacterial community, with declining bacterial diversity in treated samples (Fig. 2b). Studies on antibiotic-induced perturbations in commensal microbes have revealed similar results [31–33]. In the environmental microbiosphere, antibiotics produced by natural organisms provide mutual inhibition for competing neighbor organisms, which is vital for cell-to-cell signaling networks and maintenance of healthy species diversity [34]. The inhibitions created by naturally produced antibiotics are not intended to kill competitive bacterial, but rather to prevent undesirable overgrowth and colonization in a shared ecosystem [34]. The presence of these anthropogenic antibiotics caused asymmetrical selection on different bacterial populations. As revealed by our study, Planctomycetes, Chloroflexi, and 13 other phyla were susceptible to florfenicol, as their abundance decreased significantly after the treatment. By contrast, Proteobacteria, Bacteroidetes, Actinobacteria, and Verrucomicrobia increased dramatically after the florfenicol treatment. These results are consistent with our previous study on microbial adaptation to therapeutic oxytetracycline treatment [27], implying that the evolutionary units were pushed toward unification by antibiotic treatments. In aquaculture, a diverse and stable microecosystem plays an important role in aquaculture animal health, growth, and diet [35]. The resident microbial population protects the host from invading pathogens by competitive interaction or direct inhibition. Although no evidence has been identified in aquaculture, studies in humans have already revealed that cycles of antibiotic treatment could lead to drastically reduced fecal microbiome diversity and recurrent drug-resistant infections [36, 37]. In this study, the microbiomes of samples collected on day 25 exhibited drastic variations and reduced species diversity (Fig. 2a, b). This disturbance resulted from the florfenicol treatment may reduce microbiome-mediated colonization resistance and increase the risk of infection.

Antibiotic treatments also introduce asymmetric selection on lower evolutionary units (e.g., mobile genetic elements, genes) by acting as a strong stressor [38]. Genetic exchange communities sharing particular ARGs or beneficial mutations possess enhanced dispersal and local colonization capabilities. As revealed by this study, expanded FRGs were usually observed within microbial populations that increased dramatically after florfenicol treatment, especially for Proteobacteria and Bacteroidetes (Fig. 4). *OptrA* was the most prevalent FRG shared by over 50 microbial populations. This gene was first identified on a conjugative plasmid in *Enterococcus faecalis.* It confers resistance not only to chloramphenicol and florfenicol, but also to the oxazolidinones linezolid and tedizolid [20]. The phenicol resistance genes (*optrA*, *floR*, *cfr*, *fexA*, *cm*l, and *cat*) usually coexist with mobile genetic elements including plasmids, transposons, or integrons [19, 21]. In this study, significant correlations of ARG frequency distributions were identified within and between Genome bin (GB) populations, implying the horizontal gene transfer triggered by florfenicol treatment. In addition to phenicol resistance genes, the florfenicol treatment also induced the transmission and evolvability of genes that confer resistance to multiple drug classes, providing a resistome atlas for environmental microbials.

Frequency of mutations reflects the genetic adaption and mutation rate of a population in the process of selection [39]. In the case of antibiotic resistance, identification of SNPs facilitates tracing the resistance-conferring genes and the evolutionary counterpart of antibiotic-resistant bacterial isolates [40, 41]. As revealed by this study, overall SNP-based genetic heterogeneity did not change extensively in most populations after florfenicol treatments. Significant changes in SNP frequencies, however, were observed in multiple membrane transporters, including MFS-family permeases, ABC-type transport system permeases, and drug/metabolite transporter (DMT) superfamily permeases. Previous studies have reported that multidrug transporter systems from different permease families are involved in the efflux of phenicol [42, 43]. For instance, several permeases of the MFS family, such as Cmr and MdfA, have been reported to export phenicols from *E. coli* [44, 45]. Multidrug permeases of the RND family, such as MexAB-OprM [46] and AcrAB-TolC [47], also include phenicols in their substrate spectrums. This study’s results demonstrate that a prevalent approach to acquiring florfenicol resistance in bacteria is to promote the regulation of the microbe’s specific transmembrane transporter systems. It is noteworthy that dramatic changes in SNP allele frequencies are also observed in genes involved in genetic recombination (Fig. 5). Recombination is a crucial approach employed by bacteria to uptake and integrate exogenous DNA into the host cell genome, which allows microbes to circumvent environmental interventions or adapt to selective pressures [48]. Genetic recombination is of great importance in the development of antibiotic resistance. Antibiotic usage may induce the transformation and generation of competent cells in microbial populations, facilitating the transfer of exogenous genes that confer antibiotic resistance [49]. Studies have found that recombination events are responsible for the mosaic structure of multiple ARGs, such as genes encoding penicillin-binding proteins (PBP) in *S. pneumoniae* [50], ribosomal protection proteins (RBP) in *Megasphaera elsdenii* [51], and aph (3′)-IIa in *P. aeruginosa* [52]. Several epidemiological studies have reported that recombination mediates the distribution of transposons and integrative conjugative elements (ICEs) that carry antibiotic-resistant determinants [53, 54]. In this study, key genes involved in the RecFOR and RecBC pathway were covered by significantly changed SNPs, suggesting that variations in recombination systems may facilitate the influx of ARGs as well as the accumulation of beneficial mutations in genotypically cohesive populations.

## Conclusion

In conclusion, this study provides an in-depth understanding of the substantial impact of florfenicol treatment on microbiota in the aquaculture environment. Florfenicol treatment shifts the microbial population structure in the environmental resistome by asymmetrical selection on genes and mutations with a resistance phenotype. The comprehensive metagenomic analyses revealed the patterns and dynamics of microbial resistome and provided insight into the genetic adaptations of aquatic microbial communities after the application of antibiotics. Ultimately, information present in this study will directly help with the development of guidance for the strategic use of florfenicol.

## Materials and methods

### Catfish experimental trial setup

In this study, four 300-L aquaria were set up at Auburn University’s E.W. Shell Fisheries Research Center in Auburn, Alabama. The aquaria were filled with water and bottom sediment from a watershed reservoir and were supplied with aeration at a rate of approximately 6 ppm. To evaluate the impact of florfenicol on microbial communities in the production system, channel catfish (*Ictalurus punctatus*) of ~ 100 g per individual were stocked at a density of 25 fish per aquaria. Prior to the addition of florfenicol, catfish were fed at a typical feeding rate (~ 2.5% of body weight per day) for 2 weeks with a water flow rate of ~ 60 mL/min to establish and stabilize the microbial communities in the system. Uneaten floating feed was removed from the aquaria 30 min after feeding to avoid spoilage. Daily care and operation of the experimental system was performed as outlined in the SOP 2015-2705 which has been approved by the Institutional Animal Care and Use Committee (IACUC) at Auburn University. Commercially available antibiotic florfenicol (Aquaflor 50% Type A Medicated Article, Intervet/Schering-Plough Animal Health) was incorporated with the dry ground feed at a concentration of 2 g medicated article per kilogram prior to extrusion. Catfish received florfenicol treatment at a level of 10 mg/kg body weight and fed at a 1% body weight per day for a period of 10 days. In order to mimic an enclosed catfish pond production system, no water exchange was performed during or after the florfenicol treatment. The treatment procedure was approved by the IACUC committee at Auburn University (IACUC number: 2016-2960). After the 10-day treatment period, fish received non-medicated feed at the typical feeding level (~ 2.5% of body weight per day) for an additional 3 weeks. Water quality was measured daily (4–5 pm) throughout the experimental trial. Water temperature was 21.6 ± 2.8 °C, the pH ranged from 6.8–7.8, and the total ammonia nitrogen (TAN) concentration ranged from 0.5–3 mg/L. The un-ionized ammonia (the toxic version of ammonia that is harmful to fish) concentration was less than 0.1 mg/L, which are levels considered safe for catfish [55–57].

### Sample collection, DNA isolation, library construction, and sequencing

Water samples (1 L each) were collected from each aquarium at day 0, day 10, and day 25 after florfenicol treatment and were filtered through 0.2 um filters (EMD Millipore, Temecula, CA). The filtered membranes were then cut with scissors into small pieces for DNA extraction using a PowerSoil® DNA isolation kit (MoBio Laboratories, Carlsbad, CA) according to the manufacturer’s instructions. The library construction and sequencing were conducted at the HudsonAlpha Genomic Services Lab (Huntsville, AL, USA). Genomic libraries were prepared with the Paired-end Sequencing Sample Preparation Kit (Illumina, San Diego, CA) according to the manufacturer’s instructions. The 12 DNA libraries were sequenced on two lanes of the Illumina HiSeq 2000 platform for 100-bp paired-end reads. All the sequencing data were deposited at the NCBI BioProject repository under accession number PRJNA408155.

### Taxonomy classification and differential abundance analysis

Taxonomy classification of sequencing reads by Kaiju (version 1.4) was applied after reads trimming. Briefly, FastQC (version 0.11.5) was used to visualize the overall data quality and identify potential problems with the data. Raw reads were trimmed by removing ambiguous nucleotides (N’s), extreme short reads (< 25 bp), and low-quality bases using Trimmomatic (version 0.36). The clean reads were subjected to taxonomy classification with the representative bacterial and archaeal genomes from the proGenomes database. The abundance matrix was imported into MetagenomeSeq (version 1.6) to evaluate the sample-to-sample distances, the Shannon diversity, and the genera that are differentially abundant among samples.

### De novo metagenome assembly and gene annotation

Filtered reads from all the samples were pooled together and assembled using Megahit (version 1.1) with *k*-mer sizes from 27 to 99 with a step of 10. Contigs with a length over 2.5 kbp were organized into genome bins using MetaBat (version 0.32.4) according to the tetranucleotide sequence composition and overall contig coverage patterns retrieved from backtrack alignment files. The reads from each sample were mapped to the assembly with at least 95% sequence identity using the Burrows-Wheeler aligner (BWA)-backtrack alignment algorithm [58]. To assess the completeness of genome bins and mitigate incorrectly binning contigs from different organisms, collocated sets of ubiquitous and single-copy genes within a phylogenetic lineage were estimated in each genome bin by using CheckM (version 1.0.6). Genome bins with less than 50% completeness or more than 20% contamination were excluded from phylogenetic analysis.

The DOE Joint Genome Institute’s Integrated Microbial Genome database tool (version 4.15.1) was used to annotate metagenomic reconstructions. Briefly, open reading frames were predicted by Prodigal and GeneMark. Conserved protein families and domains were identified using BLASTP search against COG, Pfam, EC, and KEGG Orthology (KO) databases with an *e* value cutoff of 1e−10. Antibiotic-resistant genes (ARGs) were identified using the strict paradigm of Resistance Gene Identifier (version 3.2.1) which is based on the protein homolog model type sequences of the CARD database (version 1.2.0). Resistance functions of the annotated genes were also predicted according to Resfams (version 1.2) using HMMER (version 3.1b1).

### Phylogenetic analysis and taxonomic analysis of metagenomic assemblies

The taxonomic identities of identified genome bins (GBs) and their evolutionary relationships with 3171 known microbial genomes were determined using the PhyloPhlAn pipeline (version 0.99). GBs were assigned to the finest taxonomic level upon which all marker genes agreed, ranging from the phylum level for some GBs to the genus level for others. Predicted genes within each genome bin were also checked by sequence similarity to the non-redundant (NR) database using BLASTN. The taxonomic assignment of the best match generated by BLASTN was retrieved for validating results obtained from PhyloPhlAn.

### Identifying SNPs and allele frequency differences

The clean reads were mapped to the reference genomes using BWA with at least 95% identity. SNPs were identified using Varscan (version 2.3.7) with the thresholds of minor allele frequency being greater than 0.05, minimum read base quality of 20, strand-filter of 90%, and minimum read depth of 10. Allele frequencies were calculated based on the number of reads observed for the reference or alternate allele. Consistent allele frequency differences among the three sample groups were identified using Generalized Linear Models (GLMs) with quasibinomial error structure as described by Wiberg et al.’s study [59].

### Gene gain and loss

To identify genes with significantly changed relative abundance in the population over the course of this study, pairwise comparisons of gene coverage were conducted following the procedures used by Bendall et al. [60]. Briefly, gene coverage was estimated by normalizing the number of mapped reads by the gene length. Gene frequency was determined for each gene by dividing its coverage by the median coverage of all genes within a GB, which implies the copy number of each gene per cell within a microbial population. Genes were considered as gained or lost once their copy number changed by a magnitude of greater than 0.4, with a false discovery rate of less than 0.01 detected via the Metastats test [61]. The correlations of frequency changes were detected via WGCNA. Significantly correlated ARGs were clustered to construct a co-occurrence network based on the dissimilarity measure of topological overlaps [62].

## Supplementary information

**Additional file 1: Table S1.** Significantly differentially abundant phyla identified in water samples collected after the florfenicol treatment when compared with samples collected before treatment. logFC, log2-transformed fold change; FDR *P* value < 0.05 was considered significant.

**Additional file 2: Table S2.** Significantly differentially abundant genera when comparing water samples collected on day 10 and day 0. logFC, log2-transformed fold change; FDR P value < 0.05 was considered significant.

**Additional file 3: Table S3.** Significantly differentially abundant genera when comparing water samples collected on day 25 and day 0. logFC, log2-transformed fold change; FDR P value < 0.05 was considered significant.

**Additional file 4: Table S4.** Genome bins reconstructed from metagenomics-combined assembly.

**Additional file 5: Table S5.** The relative abundance of antibiotic-resistance genes on days 0, 10, and 25.

**Additional file 6: Table S6.** SNPs identified in each genome bin populations.

**Additional file 7: Table S7.** Genes covered by SNPs with significant changes of allele frequencies

**Additional file 8: Figure S1.** Heatmaps of phenicol-resistance genes with significant frequency changes. Rows of the heatmaps are clustered according to phenicol-resistance genes.

**Additional file 9: Figure S2.** COG classification of genes covered by SNPs with significant changes of allele frequencies.

## Data Availability

All the sequencing data were deposited at the NCBI BioProject repository under accession number PRJNA408155.

## Abbreviations

ARGs: Antibiotic-resistant genes
BWA: Burrows-Wheeler aligner
DMT: Drug/metabolite transporter
FRGs: Florfenicol-resistant genes
GBs: Genome bins
GLMs: Generalized linear models
IACUC: Institutional Animal Care and Use Committee
ICEs: Integrative conjugative elements
MIC: Minimum inhibitory concentration
PBP: Penicillin-binding proteins
PCA: Principal component analysis
RBP: Ribosomal protection proteins
SNP: Single-nucleotide polymorphism
