# Supplementary material for: Impacts of florfenicol on the microbiota landscape and resistome as revealed by metagenomic analysis
Source: Microbiome. 2019 Dec 9;7:155. doi: 10.1186/s40168-019-0773-8 (PMC6902485; doi:10.1186/s40168-019-0773-8)
Supplement: Supplementary file 8 — Additional file 8: Figure S1. Heatmaps of phenicol-resistance genes with significant frequency changes. Rows of the heatmaps are clustered according to phenicol-resistance genes. [file 40168_2019_773_MOESM8_ESM.pdf]

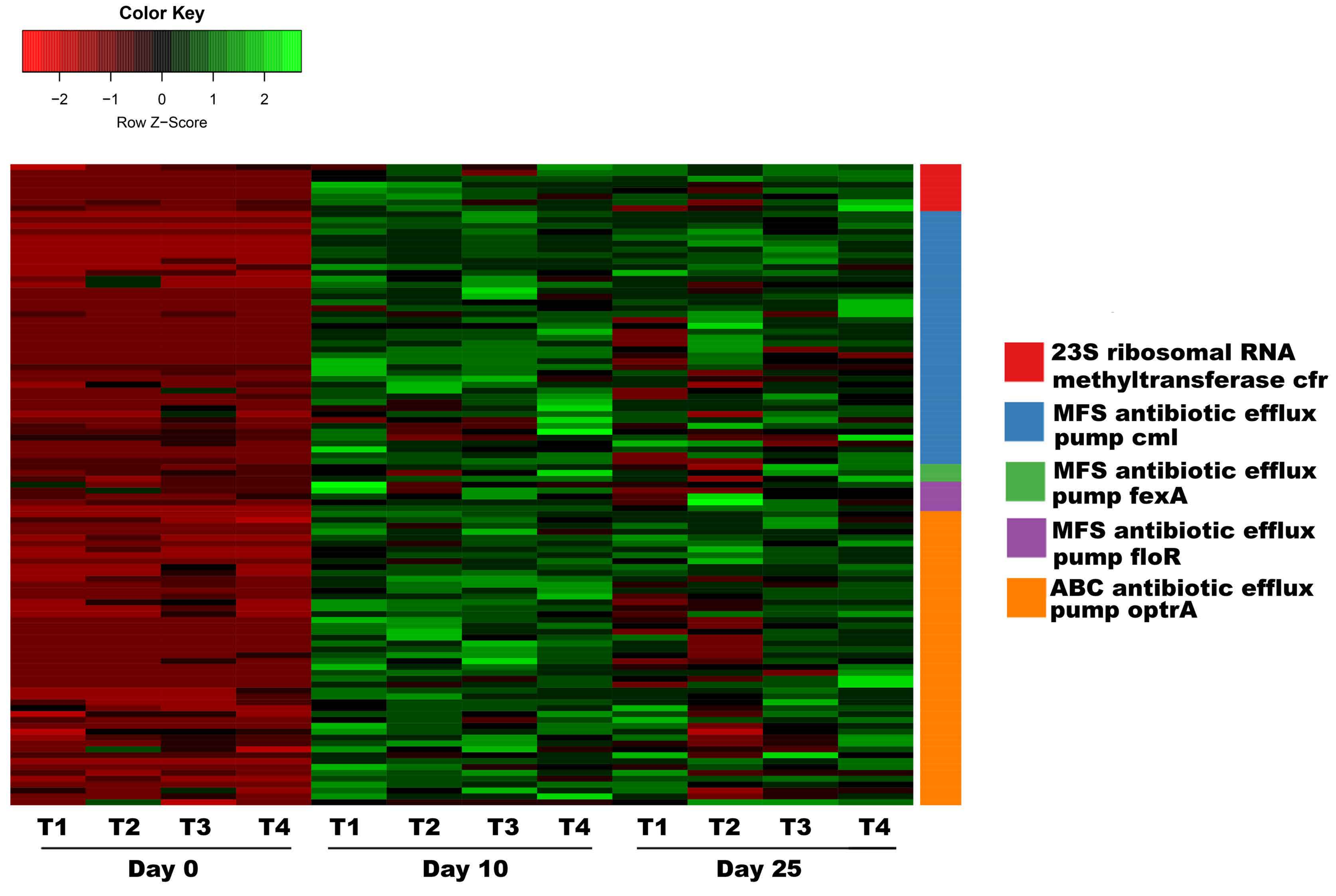

Color Key
−2
−1
0
1
2
Row Z−Score
23S ribosomal RNA methyltransferase cfr
MFS antibiotic efflux pump cml
MFS antibiotic efflux pump fexA
MFS antibiotic efflux pump floR
ABC antibiotic efflux pump optrA
T1
T2
T3
T4
Day 0
T1
T2
T3
T4
Day 10
T1
T2
T3
T4
Day 25
