# Supplementary figures and images for: Impacts of florfenicol on the microbiota landscape and resistome as revealed by metagenomic analysis
Source: Microbiome. 2019 Dec 9;7:155. doi: 10.1186/s40168-019-0773-8 (PMC6902485; doi:10.1186/s40168-019-0773-8)

## COG classification of genes covered by SNPs with significant changes of allele frequencies

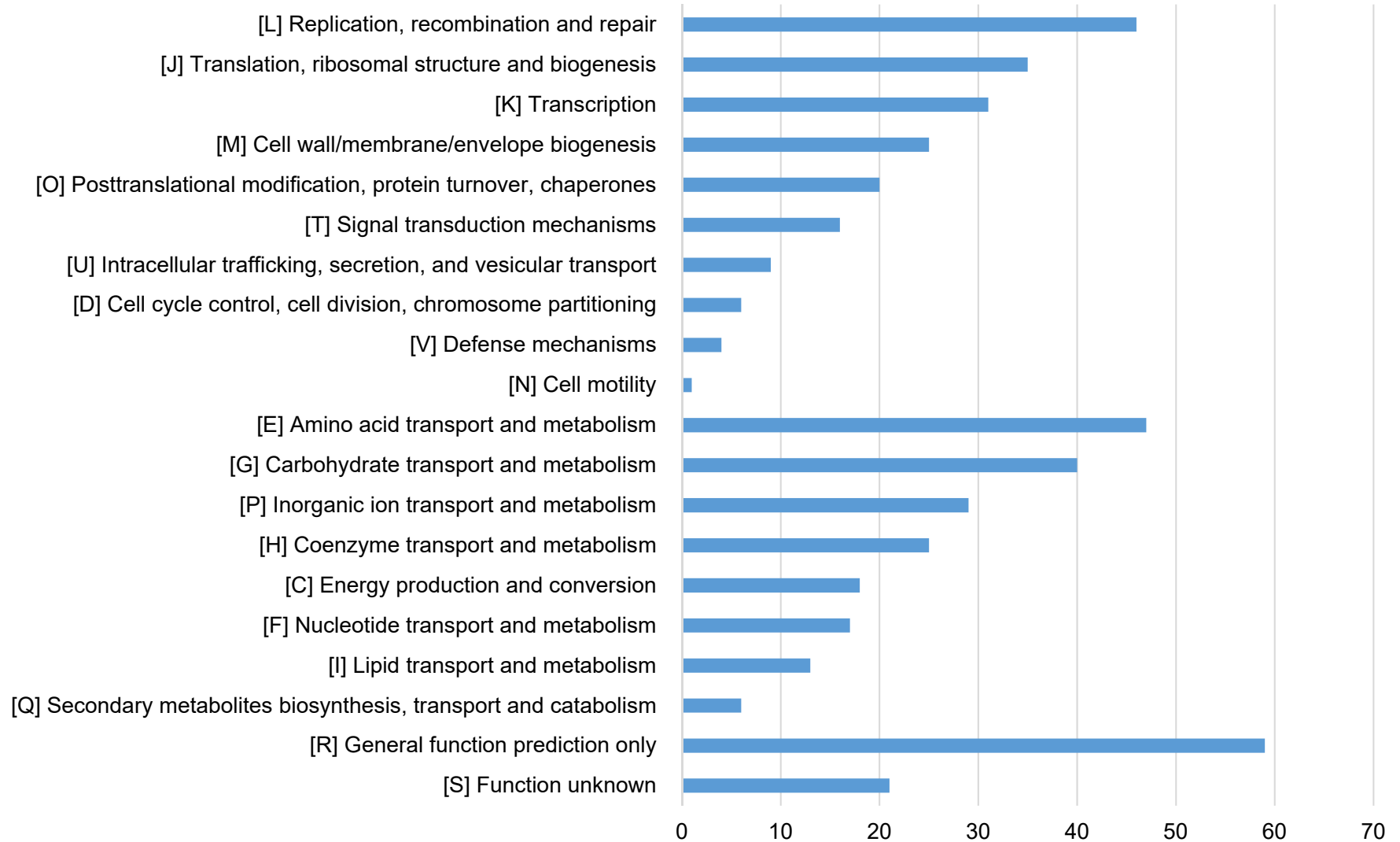

Supplement: Supplementary file 9 — Additional file 9: Figure S2. COG classification of genes covered by SNPs with significant changes of allele frequencies. [file 40168_2019_773_MOESM9_ESM.pdf]
